# Reliable biogeography requires fossils: insights from a new species-level phylogeny of extinct and living carnivores

**DOI:** 10.1098/rspb.2024.0473

**Published:** 2024-08-07

**Authors:** Søren Faurby, Daniele Silvestro, Lars Werdelin, Alexandre Antonelli

**Affiliations:** ^1^ Department of Biological and Environmental Sciences, University of Gothenburg, Box 461, 40530 Gothenburg, Sweden; ^2^ Gothenburg Global Biodiversity Centre, Box 461, 40530 Gothenburg, Sweden; ^3^ Department of Biology, University of Fribourg, Fribourg 1700, Switzerland; ^4^ Swiss Institute of Bioinformatics, Fribourg 1700, Switzerland; ^5^ Department of Palaeobiology, Swedish Museum of Natural History, Box 50007, 10405 Stockholm, Sweden; ^6^ Royal Botanic Gardens, Kew, Richmond, Surrey TW9 3AE, UK; ^7^ Department of Biology, University of Oxford, South Parks Road, Oxford OX1 3RB, UK

**Keywords:** ancestral area, Carnivora, Creodonta, extinction, historical biogeography, phylogeny

## Abstract

A central objective of historical biogeography is to understand where clades originated and how they moved across space and over time. However, given the dynamic history of ecosystem changes in response to climate change and geological events, the manifold long-distance dispersals over evolutionary timescales, and regional and global extinctions, it remains uncertain how reliable inferences based solely on extant taxa can be achieved. Using a novel species-level phylogeny of all known extant and extinct species of the mammalian order Carnivora and related extinct groups, we show that far more precise and accurate ancestral areas can be estimated by fully integrating extinct species into the analyses, rather than solely relying on extant species or identifying ancestral areas only based on the geography of the oldest fossils. Through a series of simulations, we further show that this conclusion is robust under realistic scenarios in which the unknown extinct taxa represent a biased subset of all extinct species. Our results highlight the importance of integrating fossil taxa into a phylogenetic framework to further improve our understanding of historical biogeography and reveal the dynamic dispersal and diversification history of carnivores.

## 1. Introduction

Most biogeographical studies focus exclusively on extant taxa. For some groups, this is largely unavoidable, such as in the case of birds, which have poor preservation rates and therefore a scarce fossil record [1]. Yet, for many other clades, the fossil record is sufficiently rich to inform us about biogeographic patterns and processes (e.g. [2–4]).

It may be difficult or even impossible to reliably infer the origin of clades based solely on their extant distribution and phylogeny. This is seen in many carnivore clades. An analysis relying solely on extant species, for example, inferred that Felidae (cats) were equally likely to have originated in North America or Eurasia, whereas Canidae (dogs) could have originated in North America, Eurasia or Africa [5]. By contrast, when including fossils, it becomes clear that dogs originated in North America [6], and that cats originated in Eurasia [7]. Although in those cases the fossil-based analyses did not contradict the analyses based on extant species only, they were able to increase the precision of the results (i.e. reduce uncertainty). In other cases, the fossil-based analyses can infer ancestral ranges outside the ones suggested by living taxa (e.g. [8]).

The formal incorporation of fossils into the analyses may thus be vital for obtaining reliable biogeographical inferences. However, a phylogenetic tree also contains important information that is not recoverable by a purely taxonomic, phylogeny-free reading of the fossil record. If members of a clade quickly disperse from the point of origin, the area with the oldest fossil records may simply reflect where most records occur, rather than being the location where the clade originated. For fossil taxa known from multiple regions, this bias can be corrected by explicitly incorporating sampling intensity between regions (see, e.g. [3]). While this can reduce the bias in the estimation of dispersal rates, neglecting phylogenetic relationships may lead to low precision and accuracy of inferred ancestral areas.

If dispersal is rare, a phylogenetic tree contains vital information not present in a purely taxonomic reading of the fossil record. The python snake family (Pythonidae) shows an interesting example of this. Early Miocene records of crown group pythons are known from both Africa and Australia [9,10]. This requires dispersal between Eurasia and Africa, which is relatively frequent, but it also requires over-water dispersal between Southeast Asia and Australia, which is much rarer. The directionality of this overseas dispersal, however, cannot be inferred from the family-level taxonomy alone. By contrast, phylogenetic trees—whether based on genetic data of extant taxa [11], or on total evidence data with extinct and extant taxa [12]—clearly show that pythons dispersed from Eurasia to Australia rather than in the opposite direction.

It is thus likely that neither sole reliance on the fossil record, nor phylogenetic approaches based only on extant organisms, will be optimal in unveiling the biogeographical history of clades. Instead, the most reliable results should be found through phylogenetic approaches incorporating fossils, although this is rarely done (but see, e.g. [13,14]). While previous studies have highlighted the benefit of acknowledging fossils in biogeographical analyses [15,16], we still lack a formal quantification of the effect of fossil integration in the phylogenetic inference of ancestral geographical ranges. It is also difficult to know *a priori* the consequences of sampling biases, which are widespread in the palaeontological record [17], for ancestral state reconstruction using fossils. Spatial biases in the fossil record can lead to detrimental effects in macroevolutionary analyses [18], although their effect on historical biogeography is insufficiently studied. It is, however, known that highly diverse areas are often wrongly inferred as the area of origin for groups when relying on extant taxa only [19,20]. A similar situation likely applies to analyses using all known fossil species, where ancestral areas may be disproportionally inferred for the areas with the most known fossil species. For a sufficiently biased fossil sampling, the benefits of adding a phylogenetic tree may thus potentially be counterbalanced by insufficiently acknowledging the biased record in the analysis. The relative importance of not incorporating fossils, versus potential problems of biased fossil knowledge, cannot be predicted without a set of empirically derived simulations.

Here, we estimate the impact of including fossils when inferring the biogeographical history of a clade, as compared with an analysis based solely on extant taxa. We focus on the mammalian order Carnivora and their extinct relatives Miacoidea, Hyaenodontidae and Oxyaenidae. We first reconstruct a phylogeny of all known extinct and extant species and then infer likely ancestral areas across multiple subclades. We compare these results with the outcome of analyses based only on extant species, or based solely on the fossil record but not incorporating phylogenetic information. Based on empirical results and simulations, we demonstrate that analysing fossils and extant species in a phylogenetic context produces substantially more precise and accurate results than relying solely on extant species or analysing fossils in a phylogeny-free context.

## 2. Material and methods

### (a) Phylogeny

We first revisited the taxonomy of all extant and extinct species in the study group (Carnivoramorpha, Hyaenodonta, and Oxyaenidae), to ensure that we included all species we judged to be valid. Following this, we built a phylogeny of all species in a total-evidence phylogeny using a tip-dating approach. We summarize all procedures below and provide further details on all steps in the electronic supplementary material.

#### (i) Input data

We included all extant and extinct species of mammalian carnivores and related extinct groups (Carnivoramorpha, Hyaenodonta and Oxyaenidae). Herein, we refer to this entire clade as ‘carnivores’. We revisited the taxonomy of all fossil and extant members of the group and accepted 1723 species (314 of which are extant). We based our analyses on records in the Paleobiology Database (PBDB; https://paleobiodb.org/) and the New and Old Worlds database of fossil mammals (http://www.helsinki.fi/science/now/; NOW), supplementing these with data from the original literature for 128 species that we consider valid, but which lacked any records in either of the two databases at the time of compilation. We then manually investigated the taxonomy of all names in NOW or PBDB for internal consistency and to match current knowledge (e.g. to filter out synonyms and spelling errors).

#### (ii) Phylogeny

We reconstructed the phylogeny of all extant and extinct species of carnivores using a tip-dating approach under a fossilized birth–death model in MrBayes 3.2 [21]. We did this in a two-step procedure combining a backbone tree with a number of smaller phylogenies at lower taxonomic levels. All these smaller clades were constrained to be monophyletic on the backbone phylogeny, and all but one of the species of each smaller clade were removed from the backbone before merging. Our approach therefore produced a consistent way of merging trees to generate fully dichotomous phylogenies. For a few families with short stem branches, the resulting family-level clades had crown ages slightly older than the stem ages of the overall tree in some of the trees in the posterior distribution, and we therefore needed to recalibrate the family-level trees to avoid negative branch lengths. This was done for all problematic trees (i.e. family-level trees with crown ages slightly older than the stem ages from the backbone tree) so that all branch lengths in the new tree were proportional to the branches in the original tree, and the root age was equal to the stem age of the backbone tree minus 0.01.

Our procedure is similar to that used to reconstruct phylogenies focusing on other large clades (e.g. [22,23]), but it has previously mainly been used to generate phylogenies of all extant species within a clade. The placement of species without genetic or morphological data was facilitated by a number of constraints based on taxonomy and suggested relationships from taxonomic treatments. In total 724 out of 1723 species had genetic and/or morphological data and a total of 500 constraints were employed (see electronic supplementary material, tables S1–S4). The phylogeny was reconstructed at the species level. Fossil records assigned only to genus level (for genera constrained to be monophyletic) were, however, used to determine the minimum age of the genus (the priors for the age of such genera had the age of the oldest fossil in the genus as a lower bound). These trees only give species origination times, but we combined that information with extinction times estimated by the Bayesian program PyRate [24]. These PyRate analyses were performed at the species level, unlike the PyRate analyses used to understand ancestral area (see below). The resulting phylogeny is available as Appendix 1 in Dryad (as 1000 trees from the posterior distribution of trees) [25].

### (b) Ancestral area reconstruction on empirical trees

We compared ancestral area estimated with four methods: one relying solely on taxonomy plus three phylogenetic methods. The three phylogenetic methods used the same methodology but varied in whether fossils were included or not and whether the analyses focused on stem or crown groups.

For the non-phylogenetic method, we estimated origination and extinction time for all genera using PyRate, which is a program that can explicitly account for temporal, spatial or lineage-specific biases in preservation and sampling rates [24]. We chose to conduct this analysis at the genus rather than the species level, which has both pros and cons. On the one hand, by analysing data at the genus level, we had to assume that all species within a genus had the same fossilization potential, whereas analyses at the species level could incorporate species-specific preservation rates. On the other hand, we were able to include fossil occurrences that were only identified at the genus level, which we would have to exclude if the analyses had been conducted at the species level. Many of these occurrences represent the earliest records for their genera. For this method, we inferred the ancestral area as the one having the oldest record. In all PyRate analyses (for both ancestral state reconstruction and phylogeny), we removed duplicated records from PBDB and NOW and excluded spatially and temporally overlapping occurrences (see electronic supplementary material, §S5.1.4 for more details). In short: for each species we accepted all records from the database with most records and only added records from the other database if they were not spatially and temporally overlapping with any already accepted record.

The three phylogenetic methods relied on maximum-likelihood estimations of the ancestral areas. These were inferred under both the DEC + J (dispersal–extinction–cladogenesis including jump dispersal) and the DEC (dispersal–extinction–cladogenesis) models in BioGeoBEARS [26]. There is an ongoing debate regarding which of the two models is the better for inferring ancestral areas. DEC + J is generally preferred based on Akaike information criterion (AIC) model selection, and its inclusion of founder effect speciation as a process may be more biologically realistic in some contexts [27]. While there thus are arguments for including founder speciations, the statistical validity of its implementation has been criticized (e.g. [28,29]), but see Matzke [30], and remains the subject of ongoing discussion. We therefore carried out our analyses under both models, reporting them for DEC + J in the main text, but providing all results based on DEC in the electronic supplementary material. The results were largely congruent among models, indicating that our findings are robust to the underlying biogeographical model.

We only allowed dispersal between adjoining continents (Africa/Eurasia, Eurasia/North America, and North America/South America), i.e. we set all dispersal probabilities between non-adjoining continents to zero. We allowed all species to be in a maximum of three (not necessarily contiguous) continents at once (even though most clades were found to be single-continent endemics with a very high likelihood).

We first identified ancestral areas based on the full tree. Following this, we removed all extinct species and inferred ancestral areas relying solely on extant species. We kept all species with confirmed records in the Holocene or Late Pleistocene (such as cave lions, *Panthera spelaea*, or short-faced bears, *Arctodus simus*) and considered them as ‘extant’ owing to the strong evidence for anthropogenic involvement in (nearly) all of these extinctions [31,32], thus focusing on the estimation of natural pre-anthropogenic evolutionary processes. Finally, we estimated the ancestral area of the crown group using the full tree, with the crown group defined as the most recent common ancestor of all extant species and all of its descendants. This last method was intended to evaluate whether any differences between the methods incorporating fossils and the method using extant taxa only were true differences or were due to different ancestral areas of stem and crown groups. All estimates of ancestral areas, whether based on all species or extant taxa only, were obtained from the full carnivore tree, meaning that the ancestral states of all clades were jointly estimated.

After the investigation of ancestral areas of named clades, we examined the effect of fossils across the tree. We estimated ancestral areas for the full tree, based only on extant species or with randomly sampled 5, 25 or 50% of the extinct species included in the tree. We then estimated the accuracy under the assumption that the full tree results were accurate. This was done for each of the four continents as well as a combined total accuracy. The accuracy in any given continent was defined as: Accuracy = 1 – abs(Ancestry_Full tree_ – Ancestry_Small tree_), with Ancestry_Full tree_ being the probability of the continent being part of the ancestral area based on the full tree, and Ancestry_Small tree_ being the probability of the continent being part of the ancestral area based on the tree with only some fossils included. Total accuracy was defined as the product of the continental accuracies. For this we compared all clades in the tree of extant species with the smallest possible clades in the trees with fossils containing the same species (and potentially some extinct ones as well). The analyses were conducted across 100 trees and the mean accuracy within temporal periods was calculated for each. We reported the mean of these across trees as well as the 95% highest posterior density (95% HPD) of the means across the trees.

### (c) Sensitivity to sampling biases

#### (i) Simulated trees

The comparisons so far assume that the ancestral areas were completely accurate in the full tree and that fossils are randomly sampled, but we tested both assumptions on 100 separate simulated trees with the same number of extant species as the empirical ones.

**Figure 1.**
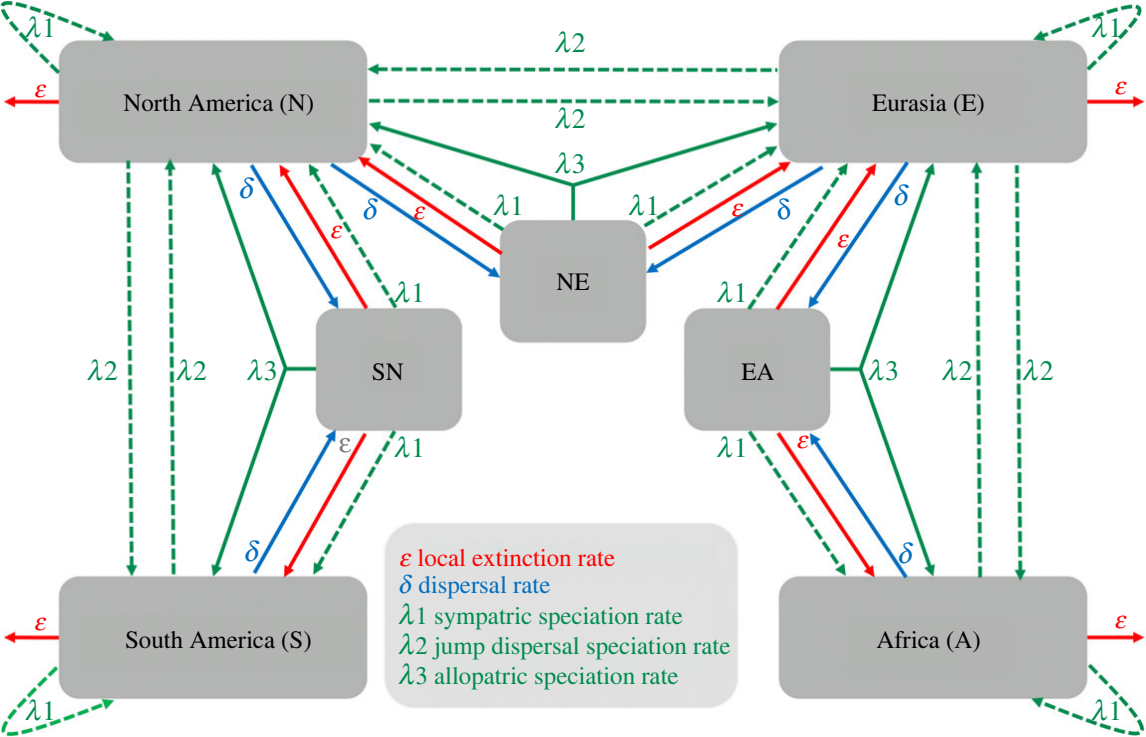
The diversification model used for this study. The large grey boxes represent major geographical regions in which species occur; the smaller boxes account for widespread species present in two of those. Arrows represent biogeographical processes within or between regions; *δ* means dispersal, *ε* extinction, and *λ* speciation (see key for details). For a full explanation of the mutational parameters see §2c.

The trees were simulated in the R package Diversitree [33] based on a modification of the GeoSSE model of Goldberg *et al*. [34]. The default GeoSSE model only contains two areas but GeoSSE models are a special case of the much more general ClaSSE model [35], and we modified a ClaSSE model to the equivalent of a four-area GeoSSE model (figure 1). Five different types of processes were included: range expansion, extinction and three types of speciation. In our model, four continents (South America, North America, Eurasia and Africa) were treated as being on a line, with dispersal only possible between adjacent regions (South America/North America, North America/Eurasia and Eurasia/Africa). Our simulations had five parameters, all assumed to be identical within the four continents: (a) the per-continent extinction rate *µ*; (b) the rate of range expansion between adjacent continents *δ*; (c) the rate of sympatric speciation *λ*_1_ (with ranges only defined at a continental level); (d) jump dispersal speciation *λ*_2_, i.e. speciation where one daughter species colonizes a neighbouring continent during speciation; and (e) the allopatric speciation rate *λ*_3_ (at the continental level), i.e. the rate where multicontinental species diversify into species endemic to each continent.

We selected plausible trees among the ones simulated by Diverstree using an ABC (approximate Bayesian computation) approach. All five diversification parameters had uniform priors (*µ* ∼ *U*(0, 1), *δ* ∼ *U*(0, 1), *λ*_1_ ∼ *U*(0, 1), *λ*_2_ ∼ *U*(0, 0.25), *λ*_3_ ∼ *U*(0, 3)). We simulated a total of 10 million trees and kept the 100 best trees based on their performance using five criteria. The criteria were: (a) empirical tree length; (b) empirical root height; (c) fraction of total species that were still extant; (d) total number of extant species found in two continents; and (e) fraction of sister species that are single-continent endemics and occur on separate continents. For (e), the extinct species were scored solely based on the last continent they went extinct on. For each simulated tree we calculated the difference from the empirical values as the absolute log error:

$$\mathrm{ALE}=\left|\log\left(\frac{r_{\mathrm{emp}}}{r_{\mathrm{sim}}}\right)\right|,$$

where *r*_emp_ is the empirical parameter value (e.g. root age) and *r*_sim_ is the simulated one. We calculated the ALE for each of the five criteria for each tree and stored the largest of the five (ALE_max_). Finally, we selected the 100 trees with the smallest ALE_max_.

#### (ii) Simulated ancestral areas

For each of the 100 trees, we ran BioGeoBEARS across the full tree, only relying on extant taxa or including biased partial sampling of fossils. We first identified empirically derived sampling rates from species-level PyRate analyses, where the rates represent the expected numbers of fossil records per lineage per million years, based on the expectation of a Poisson sampling process [24]. We then identified a factor to multiply all rates by to get a desired fraction of known fossils for each tree, and used this to estimate biased sampling across the tree. Since there is substantial temporal and spatial bias in fossilization potential, the resulting datasets will maintain similar biases. Area/time combinations with a particularly well understood fossil record, like Pleistocene North America, will thus still have the vast majority of its species known under scenarios where only a small fraction of all species are known globally.

We simulated multiple different sampling fractions and calculated the resulting accuracies. We simulated scenarios where 25, 50 or 75% of all fossils are known based on this biased sampling and then included either 5 or 25% of all fossil species in the analysis (note that both fractions refer to the fraction of all species; so, e.g. 25% included and 25% known means that all known species are included). The scenario we modelled thus simulated a scenario where only a subset of species is known (based on biased sampling), but a random number of the known fossils are included in the study. The logic for this is that inclusion in a study may be driven by a number of factors, including which museum the specimens are stored in, that are independent of sampling intensity. We then calculated accuracy in a similar way to the method for the empirical trees, except that we were comparing results to the known ancestral areas from the simulations rather than the inferred ones based on the full tree. The simulated trees had different root ages and for comparison purposes we therefore grouped them within quantiles of ages within trees rather than by absolute ages.

### (d) Predictability of accuracy

We investigated whether the reduction in accuracy of estimated ancestral ranges when omitting fossils was predictable, focusing on two potential predictors: (i) node ages, assuming that it would become progressively harder to infer the correct ancestral area without fossils the older the node is; and (ii) sampling similarity, i.e. changes in geographical distributions between extant and extinct taxa in a clade. Sampling similarity was defined as the probability that the proportions of species across continents found among extant species and among all species (extant and extinct) are samples from the same distribution based on Fisher's exact tests.

## 3. Results

### (a) Ancestral areas of named clades

**Figure 2.**
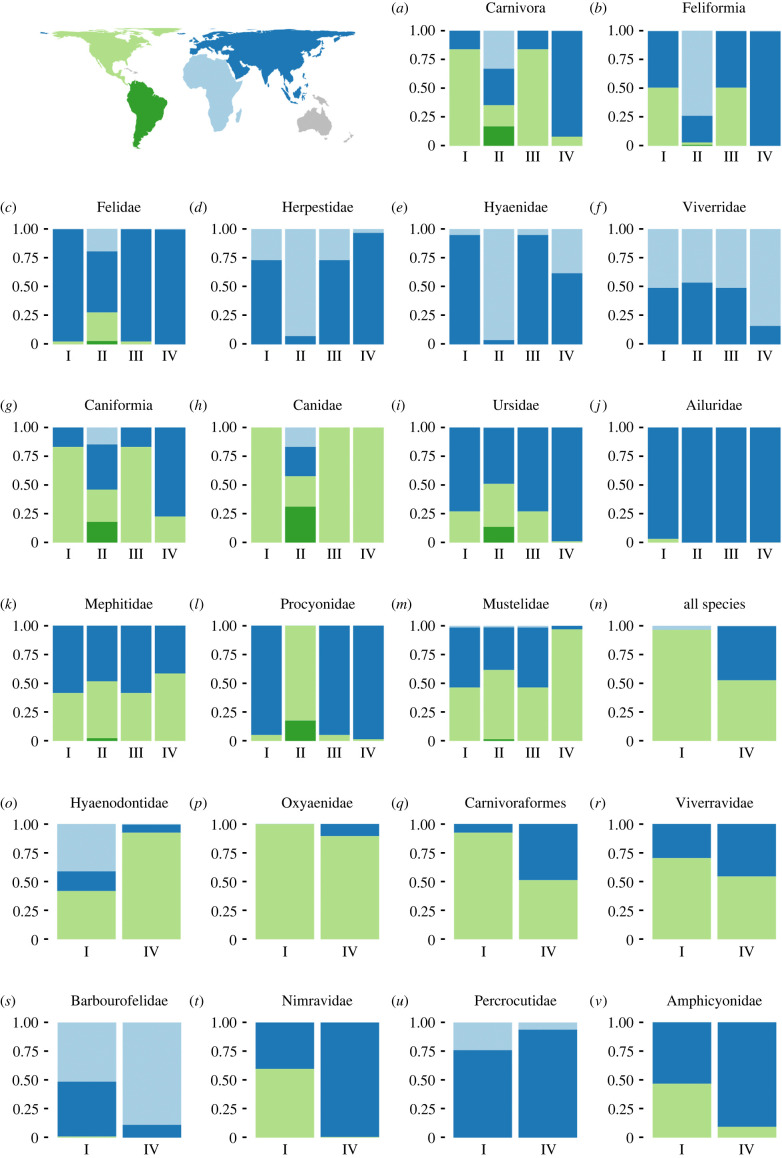
Ancestral area inferred for each monophyletic family and for selected larger well known clades in relation to method applied. The probability for each continent to be the ancestral area of each group is colour-coded as in the global map. For ten extant families within the order Carnivora, for the two suborders within it and for the order itself, we compare four methods: (I) relying on the phylogeny of all species; (II) relying on the phylogeny but removing all fossils; (III) relying on all fossils but identifying the ancestral area of the most recent common ancestor of all living species rather than the most recent common ancestor of all known species in the group; and (IV) relying only on fossil taxonomy using PyRate. Methods I–III may produce ancestral areas containing two or three continents for some clades for some trees, but the figure only shows multicontinental origin. For plotting purposes, multicontinental occurrence was divided equally among the continents (e.g. a 20% chance of being in both North America and Eurasia is plotted as 10% for being in North America and 10% for being in Eurasia). Family names in this figure and throughout the paper are used in the Linnaean sense and refer to total groups including both crown and stem species. The assignment of species to families can be seen in Appendix 3 in Dryad [25]. The figure is based on the DEC + J model. Results based on the DEC model (electronic supplementary material, figure S1) are very similar. The results above are based on averages across all 100 trees and do not account for multicontinental origins.

We identified ancestral areas for all four methods for 13 clades (figure 2) and found that the ancestral areas for the most recent common ancestor of extant species are likely to be incorrectly inferred for the majority of them unless extinct species are incorporated into the analyses. Only four of these clades had comparable results across all methods (Felidae, Ursidae, Ailuridae and Mephitidae). Two other families (Herpestidae and Procyonidae) had similar inferences for their crown group for the full tree or for the tree with only extant species but differences between these and the two other methods. The results for these two families highlight the need for precision in terminology especially when comparing patterns with and without fossils. For such comparisons it is vital to know if we are referring to crown groups (i.e. the most recent common ancestor of living species and all its descendants) or total groups, since as we see here the ancestral areas of the crown and total group can easily be different. Two clades (Viverridae and Mustelidae) had similar results for all phylogenetic methods but different results for the phylogeny-free method. The remaining five clades (Carnivora, Felifornia, Hyaenidae, Caniformia and Canidae) showed the same results for stem and crown groups based on fossils but different results in the analyses relying only on the extant species. These five are thus clearly clades where the wrong conclusions are reached if fossils are omitted from the analysis. For another nine groups, we only compared the full tree with fossils. In only three of these, did we find the same results, while the six other groups had conflicts between the results based on the two methods.

The results above are based on averages across all 100 trees and do not account for multicontinental origins. When these are incorporated, the results become slightly more complex (electronic supplementary material, figures S2 and S3). There are two main differences. Firstly, figure 2*o* suggests equal support for a North American or an African origin of Hyaenodontidae using the phylogenetic method but instead it appears that the ancestor was widespread and inhabited both continents. Secondly, for five groups where no consistent support was found for a single continental origin across the trees (Nimravidae, Viverridae, Barbourofelidae, Amphicyonidae and Mustelidae), closer inspection shows that strong support was found for many trees, meaning that clear support for one ancestral area might be retrieved if the tree could be better resolved.

### (b) Ancestral areas across the tree

**Figure 3.**
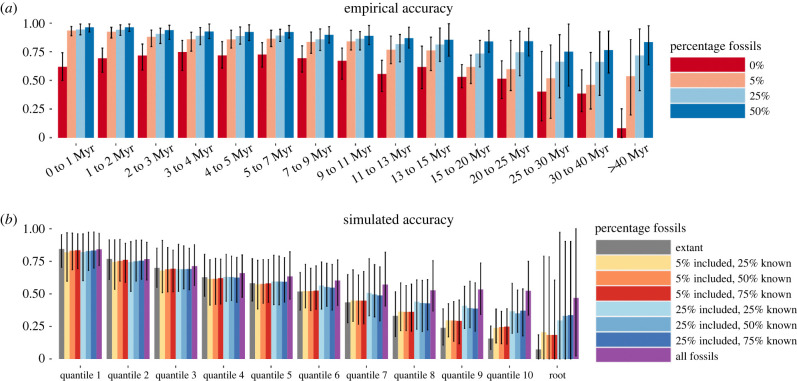
Estimated effect of adding fossils across the tree on the inference of geographical origins of clades. (*a*) Estimated effect of adding fossils across the tree based on empirical trees and random sampling. (*b*) Estimated effect of adding fossils across the tree based on simulated trees and combining biased and random sampling (see §§2b and 2c(ii) for details). For the empirical results, we define accuracy as the difference relative to an analysis using all known fossils. For the simulations, the accuracy is comparing inferred ancestral areas from BioGeoBEARS with the actual simulated ancestral areas. Both figures show results across 100 trees. The bars show means of the trees and the error bars show 95% highest posterior distributions (HPDs) of means between the trees. Only patterns in total accuracy loss are shown here. Patterns in continental accuracy and under the DEC rather than the DEC + J model (which show similar temporal patterns) are shown in electronic supplementary material, figures S4–S7.

Across the tree, we found that inclusion of fossils led to drastic improvement in the inference of ancestral areas for the empirical trees (figure 3*a*). We found that, particularly for younger nodes, the inclusion of even very small numbers of fossils led to a substantial improvement, with the majority of gains in accuracy being found after adding just 5% fossil species. More data only led to minor additional accuracy gains, which suggests that, at least for random fossils, any addition of fossil species can make a substantial difference. In this regard, we note that empirical data can only show differences and not accuracy *per se*, given that we do not have a ground truth for comparison, even though our analyses on simulated data clearly indicate that the inclusion of fossils produces more reliable results.

### (c) Simulations

Biased fossil sampling was found to be of negligible concern in our dataset (figure 3*b*). For younger nodes, we found a consistent but small decline in accuracy owing to biased inclusion of fossils, and for some cases we detected a minor decline in accuracy when including biased fossils relative to not including any. For older nodes, we found that this effect was completely overshadowed by the large benefits of including even small numbers of potentially biased fossils in the phylogeny. Even though the improvement in accuracy is only seen in older clades in the simulations, it thus seems that, for results where the addition of fossils had any meaningful effect, their addition led to more accurate results.

### (d) Predictability of accuracy

**Figure 4.**
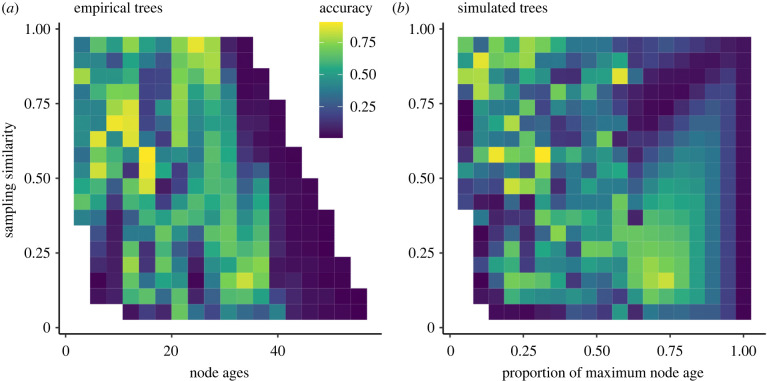
Accuracy as a function of node age (empirical trees) or proportion of maximum node age (simulated trees) and sampling similarity. Results based on DEC, which are very similar, are shown in electronic supplementary material, figure S8.

The accuracy of the inferred ancestral areas without fossils was highly correlated with both sampling similarity and node ages. Node ages and sampling similarity were too tightly correlated to be included in the same model and we therefore compared their strengths as predictors through their individual correlations with accuracy. For empirical trees under DEC + J, accuracy was more strongly correlated with sampling similarity than it was with node ages (*ρ*_Sampling similarity_ median across trees 0.58, range: 0.51, 0.69; *ρ*_Node ages_ median across trees −0.51, range: −0.59, −0.44). The difference was significant based on a paired *t*-test (*p* < 2.2 × 10^−16^). For the simulations under DEC + J, accuracy was more strongly correlated with node ages than it was with sampling similarity (*ρ*_Node ages_ median across trees −0.44, range: −0.55, −0.32; *ρ*_Sampling similarity_ median across trees 0.43, range: 0.33, 0.59). The difference was significant based on a paired *t*-test (*p* = 0.006). Through visual inspection of the results, it is however clear that both predictors matter and keeping one predictor constant shows a strong relationship between accuracy and the other predictor (figure 4).

## 4. Discussion

### (a) Ancestral areas of carnivore clades

Our results demonstrate that accurate inferences of ancestral areas require fossils, thus lending further evidence and additional perspectives to recent work highlighting the need to incorporate fossils in analyses of diversification dynamics [36]. This improvement is likely best seen in the dog family. For around 20 million years the clade was restricted to North America, where it has left a rich fossil record [6], but over the past few million years the family has colonized all other continents multiple times and its ancestral area is unrecoverable based on extant species (figure 1*h*).

Some of the cases with conflicting results between methods highlight the strength of relying on a phylogeny including fossils, rather than a simpler reading of the fossil record. This is best seen in Caniformia (figure 1*g*). Most of the oldest records of this group are North American but there are also a few old, poorly dated records of aphicyonids (beardogs) from Eurasia, which likely include the oldest record of the suborder [37]. The analysis relying only on the stratigraphic record therefore inferred the group as Eurasian in origin whereas the phylogenetic one inferred it as North American in origin. The first split within Caniformia separates Amphicyonidae from the remaining Caniformia, with the deepest split within the family separating American and Eurasian lineages, but the next two lying within Caniformia, i.e. Canidae and *Lycophocyon* are clearly North American in origin [37]. A Eurasian origin of the group therefore requires multiple migration events, whereas a North American one requires only one and is therefore more parsimonious.

Unlike the dog family—which has a very well understood fossil record—the phylogenetic placement of extinct species in many of the other families is governed by vast uncertainty. This means that the ancestral area of several of the larger groups, including Mustelidae and Viverridae, remains unknown until more information is available on the phylogenetic placement of some of the extinct forms within the families. Our analyses do, however, indicate that the ancestral area should be reliably inferable when such information becomes available, since most of the trees from the posterior distribution do suggest one specific ancestral area (electronic supplementary material, figure S2).

Most of our results are consistent with previous studies, but one result that may seem peculiar is the ancestral area for Hyaenodontidae, where all trees agree that the ancestral area comprises both Africa and North America, with some uncertainty of whether or not Eurasia is also included (electronic supplementary material, figure S2). Earlier work has suggested an African origin of the family but we note that we used a different taxonomic assignment that considers the early-diverging North American *Altacreodus* as belonging to Hyaenodontidae rather than being the sister group to the family, as in [38,39]. Two extant species (*Mustela nivalis* and *Vulpes vulpes*) are found in both North America and Africa (as well as Eurasia) and a third (*Ursus arctos*) occurred in both continents up until a few hundred years ago [40]. Such widespread distributions are thus clearly possible within carnivores, but the results still require further investigation.

### (b) Methodological considerations

Ancestral area reconstruction is expected to fail for many climatically restricted lineages. Most lineages originated under a warmer climate than today and many of these may no longer be able to live where they originated, so their true ancestral area is therefore unidentifiable by extant records only. A recent study on tree ferns [8] showed this pattern very neatly. The group originated in Laurasia, but owing to the tropical requirement of most species they are now restricted to warmer regions of Gondwanan origin. A similar claim was made in a study of ancestral areas of ants and of dragonflies, which showed that substantial differences were observed when ranges of genera were defined as all regions they are known from in fossils or extant records rather than only the current range of each genus [41].

While it can be expected that the inference of ancestral geographical range of climatic specialists is challenging to trace back in time, it could be hoped higher accuracies can be achieved for climatic generalists. Our results, however, highlight that this is not so. Carnivores are generalists in their climatic requirements, at least at the family level. For instance, species from six families of carnivores currently occur in Colombia and four of these (Felidae, Canidae, Ursidae and Mustelidae) also occur in Yukon, Canada [40]. A fifth (Mephitidae) does not occur in Yukon but did so until the end of the last ice age, and went extinct there during the megafauna extinction [42]. The sixth family (Procyonidae) does not occur in Yukon but is found as far north as northern Alberta [40]. Owing to this very wide climatic tolerance, climatic changes are unlikely to cause continental extinction at the family level. While the wide tolerance should make regional extinction at the family level less likely in carnivores than in many other groups, under some circumstances the same wide tolerance may also increase dispersal rate between regions, which could mean that ancestral areas may be harder to infer for more recent clades. In many ways, carnivores could be a near-optimal group for inferring ancestral areas relying only on extant species, at least for older clades. Despite this, we have shown that multiple errors may occur in a biogeographical inference of this clade based on extant species. We can expect similar or worse problems in many, if not most, other clades, unless fossil evidence is integrated in the analysis.

Our results give conflicting results as to whether limited addition of fossils is more important for older or younger clades. Our simulations (figure 3*b*) suggest that fossils are particularly important for older nodes, while the empirical results (figure 3*a*) suggest that fossils are particularly important for younger clades. It is not easy to interpret this apparent conflict, but we hypothesize that it is caused by complexities in the dispersal, extinction and sampling process not captured in the simple simulation model. Our simulations were carried out assuming the rate to be constant, but dispersal between continents likely became more frequent in the Pleistocene than in earlier geological time periods [3,43] and ancestral area estimation should be more difficult the more common intercontinental dispersal is.

## 5. Conclusion

We have shown that ancestral areas are frequently misidentified based on extant taxa alone. While the main take-home is that incorporating fossils is essential for increasing the reliability of biogeographical inferences, there is unfortunately no guarantee. For carnivores, we have shown that biases in the fossil record are too small to be a concern (figure 3*b*), but the fossil record for carnivores is better than for most other clades [44]. If the bias in the fossil record for a clade is sufficiently strong, the approach we have used should also produce biased results. Fortunately, the magnitude of this bias can be estimated as we have done here based on simulations using empirically derived estimates of variation in sampling. The recognition of critical gaps in knowledge arising from this could hopefully encourage further cross-disciplinary research involving taxonomists, molecular phylogeneticists and palaeontologists.

## Data Availability

All data are uploaded to Dryad Digital Repository: https://doi.org/10.5061/dryad.76hdr7t48 [25], and are referred to as appendixes 1–5. Appendix 1 contains the produced phylogeny of all carnivores. Appendix 2 contains all the R codes for the analyses. Appendix 3 contains a description of our treatment of all the individual fossil records. Appendix 4 shows topological variation across trees plotted as 18 separate subtrees focusing on smaller clades. Appendix 5 contains morphological character coding for the newly coded taxa and the MrBayes input files. Data are provided in the electronic supplementary material [45].
